# Genome Sequence Analysis and Characterization of Shiga Toxin 2 Production by *Escherichia coli* O157:H7 Strains Associated With a Laboratory Infection

**DOI:** 10.3389/fcimb.2022.888568

**Published:** 2022-06-13

**Authors:** Mark Eppinger, Sonia Almería, Anna Allué-Guardia, Lori K. Bagi, Anwar A. Kalalah, Joshua B. Gurtler, Pina M. Fratamico

**Affiliations:** ^1^ Department of Molecular Microbiology and Immunology (MMI), University of Texas at San Antonio, San Antonio, TX, United States; ^2^ South Texas Center for Emerging Infectious Diseases (STCEID), San Antonio, TX, United States; ^3^ United States (US) Department of Agriculture (USDA), Agricultural Research Service (ARS), Eastern Regional Research Center, Wyndmoor, PA, United States

**Keywords:** Shiga toxin (Stx) producing *Escherichia coli* (STEC), O157:H7, laboratory infection, genome sequencing, single nucleotide polymorphisms (SNP) typing

## Abstract

A laboratory-acquired *E. coli* O157:H7 infection with associated severe sequelae including hemolytic uremic syndrome occurred in an individual working in the laboratory with a mixture of nalidixic acid-resistant (Nal^R^) O157:H7 mutant strains in a soil-biochar blend. The patient was hospitalized and treated with an intravenous combination of metronidazole and levofloxacin. The present study investigated the source of this severe laboratory acquired infection and further examined the influence of the antibiotics used during treatment on the expression and production of Shiga toxin. Genomes of two Stx_2a_-and *eae*-positive O157:H7 strains isolated from the patient’s stool were sequenced along with two pairs of the *wt* strains and their derived Nal^R^ mutants used in the laboratory experiments. High-resolution SNP typing determined the strains’ individual genetic relatedness and unambiguously identified the two laboratory-derived Nal^R^ mutant strains as the source of the researcher’s life-threatening disease, rather than a conceivable ingestion of unrelated O157:H7 isolates circulating at the same time. It was further confirmed that in sublethal doses, the antibiotics increased toxin expression and production. Our results support a simultaneous co-infection with clinical strains in the laboratory, which were the causative agents of previous O157:H7 outbreaks, and further that the administration of antibiotics may have impacted the outcome of the infection.

## Introduction

Shiga toxin-producing *Escherichia coli* (STEC) are important food-borne pathogens. Illness usually begins as watery diarrhea, advancing to bloody diarrhea and hemorrhagic colitis (HC). Infection may progress to a serious sequela known as hemolytic uremic syndrome (HUS), which can lead to end-stage renal disease and death (Feng et al., 2022). STEC O157:H7, often referred to as enterohemorrhagic *E. coli* (EHEC), is the serotype that has most often been associated with outbreaks and severe disease; however, in recent years an increased prevalence of infections caused by non-O157 STEC serogroups has been recognized (Gould et al., 2013; NACMCF, 2019). STEC refers to those strains of *E. coli* that produce at least one member of a class of phage-encoded potent cytotoxins called Shiga toxin (Stx) or verotoxin (VT), based on cytotoxicity on Vero cells (Bergan et al., 2012). The production of Stx, as either Shiga toxin 1 or 2, or subtypes and variants thereof is a virulence hallmark of STEC. There are several subtypes within the Stx1 and Stx2 types; three are recognized for Stx1 (Stx1a, Stx1c, and Stx1d), while multiple are currently recognized for Stx2 (Stx2a through m) (Scheutz et al., 2012; Bai et al., 2018; EFSA BIOHAZ Panel et al., 2020; Yang et al., 2020a; Bai et al., 2021). Stx1a and Stx2a are the prototypes of the Stx1 and Stx2 types and are considered ‘‘wild type’’ Stx1 and Stx2 (Skinner et al., 2014). Although Stx1a has been linked to human illness, STEC that produce subtypes Stx2a, Stx2c, and Stx2d are more often associated with the development of HC and HUS (Friedrich et al., 2002; Bielaszewska et al., 2006; Melton-Celsa, 2014).

Currently, no effective prophylaxis exists for HUS (Serna and Boedeker, 2008; Goldwater and Bettelheim, 2012), and data suggest that exposure to a variety of antibiotics may increase the risk of HUS in patients infected with STEC (Mody et al., 2021; Wong et al., 2000; Tarr et al., 2005; McGannon et al., 2010; Krüger et al., 2011; Bielaszewska et al., 2012; Wong et al., 2012). The bacterial SOS-response triggered by DNA damage is linked to phage induction and consequently, an increase in Stx production (Kimmitt et al., 2000; Fadlallah et al., 2015; Krüger and Lucchesi, 2015). Several studies have shown that Stx-production is induced by the chemotherapeutic agent mitomycin C due to activation of the SOS response following DNA damage (Hull et al., 1993; Shimizu et al., 2009; McGannon et al., 2010). It has been reported that antibiotic treatment of *E. coli* O157:H7 infections is contraindicated as it has been associated with severe sequelae such as HUS (Wong et al., 2000; Serna and Boedeker, 2008). Nevertheless, some clinical studies have revealed conflicting results (Tarr et al., 2005; Smith et al., 2012), and this issue remains a controversial one (Mohsin et al., 2015). Informed by the metadata analyses of several studies the use of antibiotics in individuals with STEC infections is not recommended (Freedman et al., 2016). Variables that contribute to the development of HUS include host factors, such as age (Tserenpuntsag et al., 2005) and the characteristics of the strain involved (Grif et al., 1998; Ogura et al., 2015; Yin et al., 2015). Furthermore, STEC strains that carry the *stx*2a variant are more often associated with severe infection, and there are several subtypes of *stx*2a-phages that are associated with different toxin production levels (Ogura et al., 2015).

Shiga toxin presence and activity can be detected by Vero cell cytotoxicity, immunologic, and PCR-based assays (Gerritzen et al., 2011; Parma et al., 2012; Scheutz et al., 2012; Qin et al., 2015; He Y et al., 2016; Armstrong et al., 2018). Different immunologic Stx detection assays have shown good specificity for the different Stx types and subtypes (Parma et al., 2012; He Z. et al., 2016; Armstrong et al., 2018). PCR-based assays targeting the *stx* genes have also been used to detect specific *stx* types and subtypes (Gerritzen et al., 2011; Scheutz et al., 2012).

To investigate the source of this *E. coli* O157:H7 infection, strains that were recovered from the patient in the hospital along with strains used by the researcher in laboratory experiments were sequenced and subjected to high-resolution SNP typing. Our second major objective was to determine whether the hospital administered antibiotics may have had an impact on the Stx-expression and -production levels, and ultimately on disease severity.

## Materials and Methods

### Clinical History and Strains Used in the Study

A laboratory-acquired *E. coli* O157:H7 infection occurred in 2013 in an individual who was working in the laboratory with a mixture of six *E. coli* O157:H7 strains in a soil-biochar blend. Symptoms included bloody diarrhea, hemolytic uremic syndrome (HUS), complete kidney failure, encephalopathy, respiratory failure, and retinal hemorrhages. After symptoms developed, the researcher was hospitalized. Pulsed-field gel electrophoresis (PFGE) analysis of the two isolates recovered from the patient’s stool in the hospital and the six nalidixic acid-resistant (Nal^R^) mutant strains the researcher used in the laboratory experiment showed that the two patient isolates had indistinguishable PFGE patterns from laboratory strains *E. coli* O157:H7 strains RM7386 (7386) and RM6535 (6535). Strains used in this study, their characteristics, and sources are shown in **Table 1**.

**Table 1:** Laboratory and patient *E. coli* O157:H7 strains used in this study.

Strain	*stx_1_*	*stx_2a_*	*eae-γ1*	Source
7386 *wt*	Negative	Positive	Positive	Washington PHL strain #14873 isolated from bagged lettuce - Northwest Fruit and Produce implicated in an outbreak in Washington State in 2008
7386 *nal^R^*				
Patient isolate – M1300706001A				
6535 *wt*	Positive	Positive	Positive	Human isolate (MN) associated with an outbreak linked to iceberg lettuce in U.S. (Taco John) in 2006
6535 *nal^R^*				
Patient isolate – M1300706002				

### Genome Sequencing, Assembly and Annotation

Strains were cultured in LB broth o/n at 37°C with shaking at 180 rpm. Total genomic DNA was extracted from the o/n culture using the QIAamp DNA Mini Kit (Qiagen, Inc., Valencia, CA, USA) for Illumina MiSeq and Nanopore MinION sequencing. For PacBio RS II sequencing, genomic DNA was extracted using the Genomic-tip 500/G kit (Qiagen). To close the genomes, we pursued a hybrid approach combining short-read Illumina MiSeq, long-read PacBio RS II, and Minion (Oxford Nanopore Technologies) technologies. For Illumina sequencing, a paired-end library was prepared for all strains using the NxSeq AmpFREE Low DNA Library Kit (Lucigen) and sequenced with 250-bp read length using the MiSeq Reagent Kit v2 500-cycle (Illumina) following the manufacturer’s guidelines. For MinION sequencing, genomic DNA of *E. coli* O157:H7 strain 7386 *wt* was diluted to a concentration of 1.5 µg genomic DNA in 46 µl nuclease-free water. The library was prepared using the Ligation Sequencing Kit 1D (SQK-LSK108) in combination with the Native Barcoding Kit (EXP-NBD103) according to the manufacturer’s instructions, and sequencing was performed on a MinION Mk1B. MinION reads were assembled with Canu version 1.1 (Koren et al., 2017). In addition, PacBio long-reads were generated at the University of Delaware DNA Sequencing and Genotyping Center, Delaware Biotechnology Institute in Newark, DE and at the Drexel University Genomics Core Facility in Philadelphia, PA. Genomic DNA was sheared into approximately 10-kb fragments using g-TUBE (Covaris, Inc., Woburn, MA, USA). The library was prepared based on the 10-kb PacBio sample preparation protocol and sequenced using P6/C4 chemistry on four single-molecule real-time (SMRT) cells with a 180-min collection time. The continuous long-read data were assembled *de novo* using the PacBio hierarchical genome assembly process (HGAP version 2.3.0) (Chin et al., 2013). Contigs were merged and circularized using Circlator (v 1.0.2). Assemblies were polished, and motifs were detected using RS_Modification_and_Motif_Analysis (v 2.3.0). The integrity of the resulting PacBio assemblies was evaluated by Canu (v 1.1) assembly of the MinION (Koren et al., 2017) reads for strain 7386WT and further by using Illumina short-reads in combination with the available Nanopore and/or PacBio long-reads with the hybrid assembler Unicycler (Wick et al., 2017) with Pilon error correction (Walker et al., 2014). Finally, molecules were rotated to the *oriC* (Luo and Gao, 2019) or *repA* genes for the chromosome and pO157 plasmid and annotated using the NCBI Prokaryotic Genome Annotation Pipeline (PGAP) (Tatusova et al., 2016).

### Identification of Virulence/Resistance Genes and Shiga-Toxin and Intimin Subtypes

Virulence and resistance genes were identified *in silico* with VirulenceFinder (Joensen et al., 2014; Kleinheinz et al., 2014; Joensen et al., 2015), VFDB (Chen et al., 2016) and Card (Alcock et al., 2020). To determine the Shiga toxin subtype, a single colony from each strain was selected from tryptic soy agar plates and grown in Luria Bertani medium overnight (o/n). DNA template was prepared by incubating 100 μl of the bacterial culture in 900 μl of sterile H_2_O at 100°C for 10 min. PCR assays to identify *stx* subtypes were performed according to Scheutz et al. (2012) using a ProFlex PCR system (Thermo Fisher, Waltham, MA, USA) with slight modifications as indicated in Baranzoni et al. (2016). The assays targeted *stx*2a, 2b, 2c, 2d, 2e, 2f, and 2g. Gel electrophoresis using 1 µl of amplified DNA was performed using 2.0% UltraPure Agarose (Invitrogen, Carlsbad, CA, USA) with 0.5X GelRed (Phenix Research Products, Candler, NC, USA) in 1X Tris-acetate-EDTA buffer at 100 V for 1 h, and products were visualized using an AlphaImager gel documentation system (Alpha Innotech, San Leandro, CA, USA). The allelic subtype of the intimin (*eae*) was determined *in silico* according to Lacher et al. (2006) and Yang et al. (2020b).

### Core Genome SNP Discovery

The core genome SNP discovery pipeline is implemented on Galaxy (Goecks et al., 2010; Afgan et al., 2018), an open-source web-based bioinformatics platform. The SNP discovery strategy, detailed in Rusconi et al. (2016), allows to determine strain-to-strain variation and to establish phylogenetic relationships within the genomes of various microbial pathogens (Eppinger et al., 2010; Eppinger et al., 2011; Eppinger et al., 2014; Nicholson et al., 2020; Petro et al., 2020). The core genome is defined as the set of genic and intragenic regions that are not repeated, do not contain phages, IS elements, plasmid regions, genomic islands, or other mobile genetic elements, which evolve at different rates and are not indicative of evolutionary relationships. These regions were determined for the complete annotated reference *E. coli* O157:H7 strain EC4115 chromosome (CP001164.1) as follows: repeats with NUCmer (Delcher et al., 2003) by running the reference against itself to find repeated regions, prophages with PHASTER (Zhou et al., 2011; Arndt et al., 2016; Arndt et al., 2017), IS elements using ISEScan (Xie and Tang, 2017) in Galaxy (Afgan et al., 2018), and plasmids using PlasmidFinder (Carattoli et al., 2014). The SNP discovery and verification pipeline contains the following modules:

#### SNP Discovery and Typing

Illumina reads for all six strains were uploaded in Galaxy for read-based SNP discovery. First, reads were aligned with BWA-MEM (Li and Durbin, 2009) to the selected reference genome EC4115. Resulting alignments were processed with Freebayes (Garrison and Marth, 2012) with the following threshold settings: mapping quality 30, base quality 30, coverage 10, and allelic frequency 0.75. Assemblies were analyzed using the contig-based workflow. Genomes were aligned with NUCmer against the reference strain EC4115 and SNPs were called with delta-filter and show-snps distributed with the MUMmer package (Delcher et al., 2003). The resulting SNP panel for each of the query genomes was used for further processing.

#### SNP Curation

To account for false positive calls, we used several SNP curation strategies detailed in our previous works (Eppinger et al., 2011; Eppinger et al., 2014; Rusconi et al., 2016; Nyong et al., 2020): SNPs located within repetitive or mobile regions in the reference (repeats, bacteriophages, plasmids and/or IS elements) were excluded as previously described (Eppinger et al., 2011). SNPs were further curated by extracting the surrounding nucleotides (40 nt) for each predicted SNP in the reference genome and BLASTn of this fragment against the query genomes (Altschul et al., 1990). Finally, resulting alignments were parsed to remove SNP locations with missing information (“no hits”), SNPs derived from ambiguous hits (>=2), low alignment quality or misalignments, non-uniformly distributed regions, and InDels, as previously described (Eppinger et al., 2011; Rusconi et al., 2016). Multinucleotide insertions and deletions of polymorphic bases were not considered SNPs and were excluded

#### SNP Annotation and Distribution

The curated catalogued SNPs from each query genome were merged into a single SNP panel, hereby reporting the SNP position, allelic and genic/intergenic status, and annotation.

### SNP Validation of *In Silico* Predicted SNPs

To confirm the *in silico* predicted SNPs linked to nalidixic acid resistance (Saenz et al., 2003; Fabrega et al., 2009), we performed Sanger amplicon sequencing of the DNA gyrase and topoisomerase IV genes in clinical strain pairs 7386Nal/M1300706001A and 6535Nal/M1300706002 (Genewiz). Primers were designed with Primer Express (Applied Biosystems) and are listed along with PCR cycling conditions in **Supplementary Table 1**. Each reaction was performed in a volume of 20 μl using the Phusion High-Fidelity PCR Master Mix (Thermo Scientific) followed by PCR product purification with GeneJET PCR Purification Kit (Thermo Scientific), prior to Sanger sequencing (Genewiz). To confirm SNP alleles, sequencing results were compared to the corresponding SNP positions in the reference strain EC4115.

### Phylogenomics

#### SNP Based Phylogeny

The identified curated SNP panel was used for phylogenetic reconstruction by maximum parsimony with PAUP v4.0a136 (Wilgenbusch and Swofford, 2003) with a 100 bootstrap replicates. The SNP tree was visualized in Geneious (vR9) (Kearse et al., 2012) and the majority consensus tree was built in Mesquite (Maddison and Maddison, 2021) and decorated with Evolview (Zhang et al., 2012; He Z. et al., 2016). Calculation of the consistency index for each SNP allowed us to identify parsimony informative SNPs and flag homoplastic SNPs as described in our previous works (Rusconi et al., 2016; Nyong et al., 2020).

#### Whole Genome Alignment-Based Phylogeny

To establish a phylogenetic framework and position the laboratory- and patient strains in the broader context of the O157:H7 step-wise evolutionary model (Wick et al., 2005; Feng et al., 2007), we constructed a whole genome phylogeny, including representative isolates for each of the nine clades (Manning et al., 2008) (**Supplementary Table 2**). The phylogeny was inferred by whole genome alignment with Mugsy (Angiuoli and Salzberg, 2011) and RAxML with 100 bootstrap replicates (Stamatakis, 2014). The tree topology was visualized in Geneious (Kearse et al., 2012) and decorated with respective strain-associated metadata in Evolview (Zhang et al., 2012; He Z. et al., 2016).

### Minimum Inhibitory Concentration (MIC) and Antibiotics-Induced Stx2 Production

#### Determination of the Concentration of Antibiotics to Assess Their Effect on Toxin Production

The antibiotics tested on the strains were the same as those used in the treatment of the patient. Patient treatment consisted of a combination of 500 mg of metronidazole (Flagyl, MET) and 250 mg of levofloxacin (Levaquin, LEV) each day for a total of 16 days. MET was administered more than once on a number of days, and LEV was administered 3 times on one of the days. The expected peak plasma concentrations (Cmax) for MET at the treatment dose are expected to be ~25 µg/ml [Mandell et al., 2005; National Center for Biotechnology Information, PubChem Compound Database: http://pubchem.ncbi.nlm.nih.gov/compound/metronidazole (metronidazole C6H9N3O3)], and this concentration was used for testing the bacterial cultures since this was just below the assessed MIC (Andrews, 2001). For LEV, the expected Cmax indicated in the literature is approximately 5.0 µg/ml (range 4.1 to 11.3 µg/ml) at the dosage used in the patient (Sowinski et al., 2003; National Center for Biotechnology Information, PubChem Compound Database: http://pubchem.ncbi.nlm.nih.gov/compound/levofloxacin [C18H20FN3O4]). However, a concentration of 5.0 µg/ml killed the bacteria, and thus, 50 ng/ml (just below the MIC**)** was used. MET (RPI Research Products International, Mount Prospect, IL) and LEV (Chem-Impex International, Inc., Wood Dale, IL) were tested alone and in combination. As a positive control, mitomycin C (MMC), (Millipore Sigma, St. Louis, MO) which induces production of Stx2 was used at a concentration of 50 ng/ml, and tryptic soy broth (TSB) (Becton Dickinson, Franklin Lakes, NJ) without antibiotics was used as the negative control. The experiments were performed in duplicate and repeated twice.

#### Determination of Stx2 Levels in Strains Exposed to Antibiotics

Single colonies of each strain were inoculated into 10 ml of TSB and grown overnight at 37°C at 150 rpm, and then diluted 1:50 into fresh TSB and TSB with different antibiotics (2.0 x 10^7^ CFU/ml in 10 ml TSB) (Skinner et al., 2014) and incubated at 150 rpm for 18 h at 37°C. The TSB was supplemented with either 50 ng/ml MMC, 25 μg/ml MET, or 50 ng/ml LEV, and with both 25 μg/ml MET and 50 ng/ml LEV. After incubation of the strains with the antibiotics for 18 h, the cultures were diluted 10-fold in TSB and plated onto tryptic soy agar (TSA), and colonies were counted to determine the CFU/ml after exposure to the antibiotics. The cultures were then centrifuged at 5,000 × *g* for 15 min at 4°C, and the supernatants were sterilized using 0.2-μm filters. Subsequently, the sterile supernatants were used for the quantification of Stx2 amount using a commercial indirect ELISA kit (Eurofins-Abraxis, Warminster, PA, USA; (PN 542010; https://abraxis.eurofins-technologies.com/home/products/rapid-test-kits/bacterial-toxins/shiga-toxin-elisa-plates/shiga-toxin-2-stx-2-elisa-96-test/) as recommended by the manufacturer. The sensitivity of this method is 30 pg/ml. Positive and negative controls were those included in the kit. For quantification of Stx2 production, a standard curve was simultaneously generated with each assay using serial dilutions of purified lyophilized Stx2a toxin from *E. coli* (List Biological Laboratories, Campbell, CA, USA; https://www.listlabs.com/products/shiga-toxins). A linear production was observed when serial dilutions included concentrations from 2.5 ng/ml to 78 pg/ml. Samples were diluted until their ODs were included in those shown in the standard curve to be in the linear range; most samples required a 1:500 dilution. The results were analyzed spectrophotometrically at 450 nm using a TECAN Safire II plate reader (Tecan, Morrisville, NC). The experiments were performed in duplicate and repeated at least twice.

### Reverse Transcription (RT)-qPCR to Determine Expression of *Stx*2a

Expression of the *stx*2a gene in the laboratory and clinical strains was quantified using a RT-qPCR assay under non-induced, MMC, and antibiotic-induced conditions. A total of six strains were grown from an overnight culture in Luria-Bertani broth (LB, Thermo Fisher Scientific) at 37°C and 180 rpm to an OD_600_ of 0.3-0.5. Cultures were induced either with MMC at 0.5 µg/ml (Allué-Guardia et al., 2014) or a combination of the two hospital administered antibiotics, MET and LEV, at concentrations of 25 µg/ml and 50 ng/ml. After cultures were incubated for 18 h at 37°C at 180 rpm, total RNA was extracted using the PureLink RNA Mini Kit (Thermo Fisher Scientific) and treated with DNAse I (DNAse I, amplification grade, Invitrogen) following the manufacturer’s instructions. The RNA quantity and integrity were assessed at an absorbance of 260 nm using a UV-spectrophotometer and by agarose gel electrophoresis. RNA was then converted into cDNA (RevertAid H minus first strand cDNA Synthesis kit (Thermo Scientific), and *stx*2a was quantified using the Go-Taq^®^ qPCR Master Mix (Promega, Madison, WI) and MicroAmp Fast optical real-time 96-well PCR plates (Applied Biosystems, Grand Island, NY) on an ABI StepOne PLUS Real-Time PCR system (Applied Biosystems). The primers and the cycling program used are described in **Supplementary Table 1** (Wang et al., 2002; Gobert et al., 2007). Specificity was checked by analyzing the melting curves. Expression levels of *stx*2a were normalized against the endogenous gene *tufA* using the ABI StepOne PLUS System SDS software (Applied Biosystems) and are shown in relative quantity (RQ). Two biological replicates were conducted for each strain tested.

### Statistical Analyses

Statistical significance was determined by two-way analysis of variance ANOVA using Prism (version 9.0.1) (GraphPad Software, San Diego, CA) for comparison of Shiga toxin production and expression among the different culture conditions within each strain and among the different strains for each culture condition. Differences among groups were performed by Tukey’s multiple comparison test. Kruskal Wallis-non-parametric ANOVA and Dunn’s multiple comparison test were used for analysis of log CFU/ml results. Statistical significance was considered when *p* < 0.05. A confidence level of 95% was applied.

## Results and Discussion

### Laboratory-Associated Infection With *E. coli* O157:H7

A researcher working with strains of *E. coli* O157:H7 developed sharp elbow pain with flu-like myalgias, followed by pain in multiple joints and eye pain two days after conducting an experiment with a six-strain cocktail of Nal^R^ *E. coli* O157:H7. His illness progressed to bloody diarrhea and severe abdominal pain that was alleviated with hydromorphone. Assessment following hospital admission was to refrain from antibiotic use until it was determined that infection was not due to STEC. Nonetheless, on the same day of admission, serologic testing for *Campylobacter* using the ImmunoCard STAT!^®^CAMPY assay returned a positive result. The patient was then started on two cycles of LEV and MET on days 1 and 2, before laboratory tests indicated the presence of STEC. The antibiotics were stopped on day 3 and then recommenced on day 4 for a total treatment time of 16 days, and the patient was diagnosed with pancolitis.

The *E. coli* O157:H7 infection was reported to the PA State Health Department where stool samples were sent for confirmation. The patient’s clinical course continued to deteriorate, and one week after hospitalization, the patient developed HUS, followed by respiratory failure, encephalopathy with seizures, and retinal hemorrhages. After treatment with hemodialysis and plasmapheresis the condition gradually improved and the individual was discharged 24 days after admission. The researcher was working with a mixture of a total of six STEC strains in the laboratory. The two strains isolated from the patient had indistinguishable PFGE patterns to two laboratory strains 6535 and 7386 that were part of the STEC strain mixture, pointing to a likely source of the patient’s severe O157:H7 co-infection**.** These clinical isolates are linked to an outbreak caused by contaminated iceberg lettuce and bagged lettuce, respectively (**Table 1**). Both strains carried the loci encoding the *stx*2a subtype and the *eae-γ1* gene. The virulence profiles are shown in **Supplementary Table 3**, neither strain encodes antibiotic resistance genes. Cases of laboratory-associated infections caused by *E. coli* O157:H7 have been reported (Booth and Rowe, 1993; Burnens et al., 1993; Rao et al., 1996; Coia, 1998; Salerno et al., 2004; Spina et al., 2005). The latter article describes four laboratory-associated cases that occurred in New York State from 1999 to 2004. The authors stated that standard laboratory biosafety practices had not been strictly followed, and in the four cases, the low infectious dose and ability of the pathogen to survive on surfaces for prolonged periods contributed to transmission. Regular assessment of laboratory safety protocols, adequate training, and ensuring compliance are important to avert exposure to potential hazards and for prevention of laboratory-acquired infections.

It has been reported that stressing *E. coli* O157:H7 with quinolone antibiotics such as LEV can induce a bacterial stress response due to perturbation of the DNA gyrase (topoisomerase IV) cascading to a Gram-negative “SOS response” (Nassar et al., 2013), leading to increased production and release of Shiga toxin 2 (Stx2) and escalating the likelihood of hemolytic uremic syndrome (Wong et al., 2000; Panos et al., 2006; McGannon et al., 2010; Smith et al., 2012; Wong et al., 2012). However, treatment with certain classes of antibiotics does not result in an increase in Stx production (McGannon et al., 2010; Bielaszewska et al., 2012). McGannon et al. (2010) reported that antibiotics that interfere with DNA synthesis, including ciprofloxacin and trimethoprim-sulfamethoxazole, increased Stx production, but other classes of antibiotics that target the cell wall, ribosome, or RNA polymerase did not. Antibiotics (LEV and MET) were administered to the patient described in this report due to a positive result with the stool antigen test for *Campylobacter*. LEV is a quinolone that interferes with DNA replication, and MET belongs to the nitroimidazole class and inhibits nucleic acid synthesis. The stool antigen test for *Campylobacter* used on the patient has been reported to have a positive predictive value (PPV) of only 36.6% (Fitzgerald et al., 2016). Later it was however determined that the patient was not infected with *Campylobacter*. This conclusion was based on a negative PCR result testing the patient’s stool for *Campylobacter*, thus the initial *Campylobacter* antigen test gave likely a false positive result. A similar incident occurred in June of 2013 in Virginia when a 69-year-old woman infected with STEC O111 was misdiagnosed with *Campylobacter* by the same immunoassay used on the patient in the current report (Operario et al., 2014). The woman was treated with azithromycin, ciprofloxacin, and metronidazole, and she subsequently developed hemolytic uremic syndrome. The authors stated that *“Campylobacter was not confirmed by culture or PCR, suggesting that the initial Campylobacter EIA* [enzyme immunoassay] *was likely a false-positive result.”*

### Whole Genome Sequence Typing (WGST) and Phylogenomic Relationship of Patient Recovered Isolates

**Figure 1:**
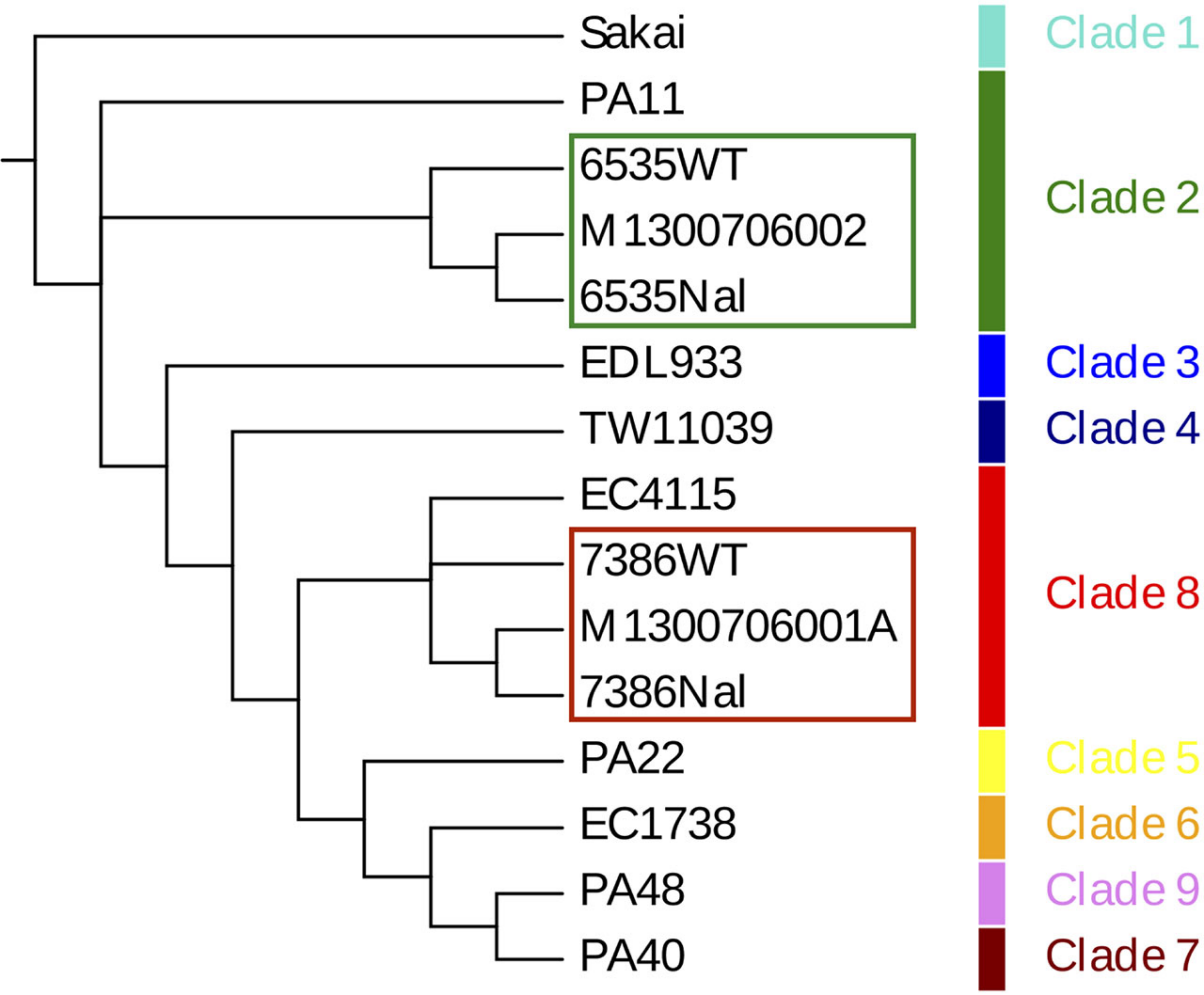
Whole genome phylogeny of representative EHEC O157:H7 strains Genomes of a total of 15 *E. coli* strains were aligned using Mugsy (Angiuoli and Salzberg, 2011) and included *wt*/NalR laboratory strains 7386 and 6535 and two clinical strains recovered from the patient and were complemented by representative isolates in the stepwise evolutionary model of EHEC O157:H7 (Feng et al., 2007; Manning et al., 2008).The phylogenetic tree was constructed using RAxML (Stamatakis, 2014) with 100 bootstrap replicates and decorated with the strain-associated metadata in Evolview (He Z. et al., 2016). Bootstrap values below 100 are shown in the tree.

**Figure 2:**
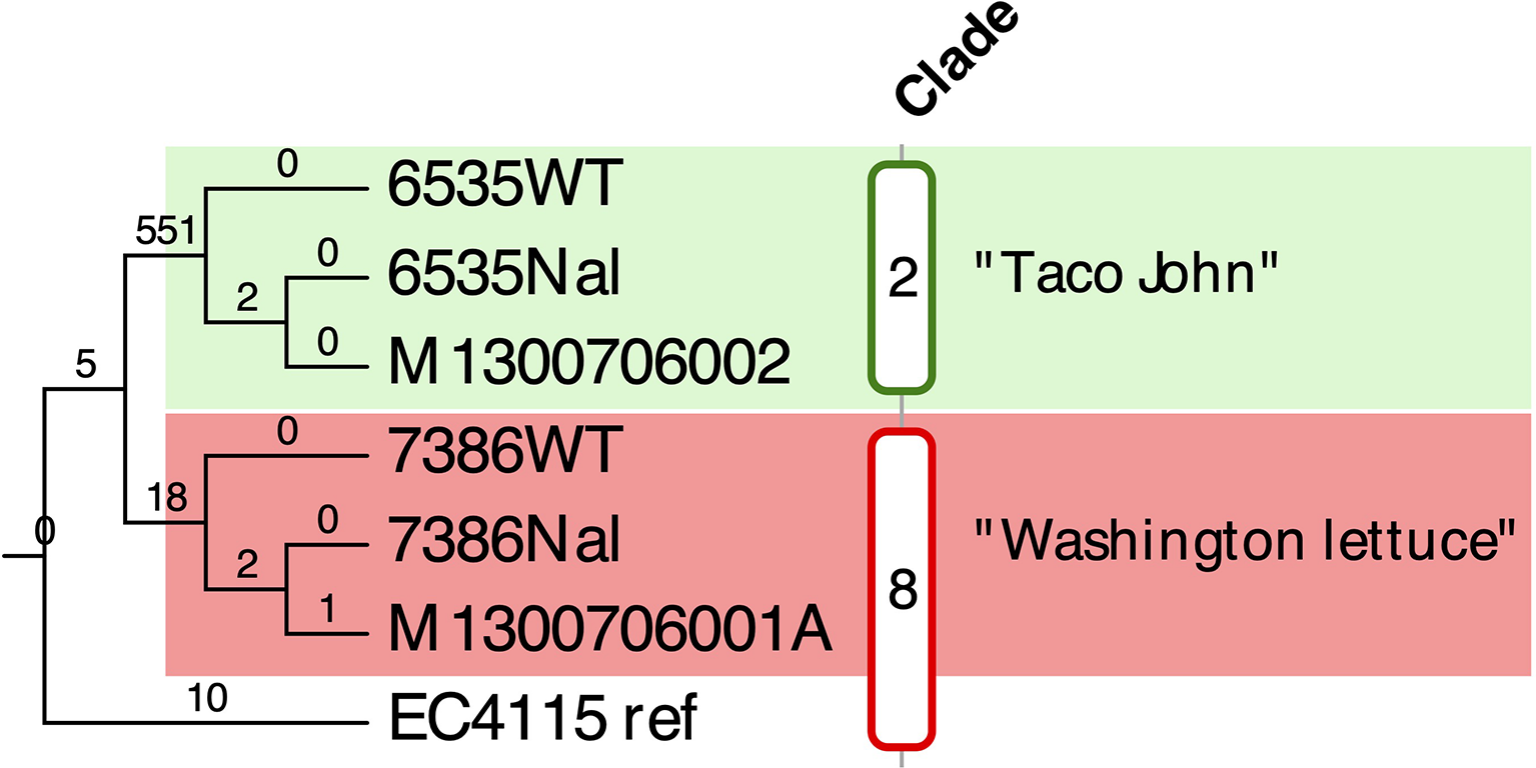
Maximum parsimony (MP) SNP-based tree of analyzed laboratory and clinical EHEC O157:H7 strains Comparison of the six genomes yielded a total of 588 SNPs of which 572 were parsimony informative. The tree shown is a majority-consensus tree of 100 equally parsimonious trees with a consistency index of 0.998 and was recovered using a heuristic search in Paup 4.0a163 (Wilgenbusch and Swofford, 2003). The phylogeny revealed two distinct clusters comprised of laboratory strains 7386 and 6535 and respective derived NalR mutants and clinical isolates recovered from the patient. The SNP tree unambiguously identified the two laboratory strains as source of this life threatening O157:H7 co-infection. As evidenced both patient recovered strains form tight clonal clusters with their respective laboratory progenitor strains and the tree topology unambiguously identified the laboratory strains as progenitor and thus source of the researchers’ infection. Only nodes with bootstrap values below 100 are listed.

Our phylogenomic analyses revealed how the analyzed laboratory- and patient strains fit into the phylogenomic context of the O157:H7 lineage. Numerous genomic epidemiology studies of STEC have embraced whole genome sequence typing (Franz et al., 2014; Sadiq et al., 2014; Eppinger and Cebula, 2015; Pightling et al., 2018), which proved critical for strain attribution and source identification (Bono, 2009; Eppinger et al., 2011; Holmes et al., 2015; Strachan et al., 2015; Yin et al., 2015; Cowley et al., 2016; Lupolova et al., 2016; Lee et al., 2017). To further investigate the source of infection, we sequenced a total of six genomes associated with this clinical case using NGS short- and long-read technologies on the Illumina, Oxford Nanopore, and PacBio platforms. The two isolate sets were comprised of *wt* outbreak strains 7386 (Washington lettuce, clade 8) and 6535 (Taco John, clade 2), the two derived Nal^R^ mutant strains the patient was exposed to in the laboratory, and further the two isolates recovered from the patient. To investigate the genetic relatedness of the *wt* and respective derived Nal^R^ mutants and clinical strains recovered from the patient, we reconstructed a phylogenomic hypotheses inferred from whole genome alignment (**Figure 1**). We included representative isolates from the stepwise evolutionary model of O157:H7, which has been refined by many groups (Whittam et al., 1988; Wick et al., 2005; Feng et al., 2007; Leopold et al., 2009; Zhou et al., 2010; Jung et al., 2013). The tree topology partitions the isolates into nine distinct clusters, as previously established by Manning (Manning et al., 2008). As evident from the tree topology, the Taco John (6535) and Washington lettuce outbreak (7386) strains, along with their laboratory-derived Nal^R^ mutants and isolates recovered from the patient, cluster with the representative clades 2 and 8 strains PA11 (Hartzell et al., 2011) and EC4115 (Eppinger et al., 2011), respectively. It is in the nature of isolates linked to single outbreaks to form tight clonal clusters and consequently outbreak investigations require the application of high-resolution subtyping strategies (Eppinger et al., 2011; Rusconi et al., 2016). To increase resolution and resolve the intimate relationship of the strains, we applied high resolution SNP typing (**Figure 2**). Core genome SNPs are highly informative in the context of outbreak investigation to differentiate “near clonal” outbreak isolates in support of strain attribution and outbreak ex- and inclusion (Eppinger et al., 2014; Jenkins et al., 2015; Rusconi et al., 2016). Whole genome SNP discovery and typing provided the necessary resolution to resolve the genetically homogenous population structure and allowed to differentiate near clonal laboratory *wt* and Nal^R^ mutant and clinical strains. The SNP analysis yielded a total of 588 SNPs, of which 572 were parsimony informative. Phylogenetic reconstruction based on the curated high-quality SNP panel with PAUP (Wilgenbusch and Swofford, 2003) identified two genetically distinct clusters separated by 551 SNPs, each comprised of the respective laboratory *wt* strains, 7386 and 6535, derived Nal^R^ mutants, and clinical isolates recovered from the patient. Strains within clusters 7386 and 6535 are near clonal and are either indistinguishable on the SNP-level or separated by one or two SNPs (**Figure 2** and **Supplementary Table 4**). The Nal^R^ mutants feature non-synonymous SNPs in both the DNA gyrase and topoisomerase genes, which are known mutations conferring this particular resistance phenotype (Saenz et al., 2003; Fabrega et al., 2009). The *in silico* predicted SNPs for these two genes were further confirmed by Sanger amplicon sequencing. In STEC O157:H7 we previously demonstrated that such numbers of SNPs can arise during a single passage in the laboratory (Eppinger et al., 2011). The recorded SNP numbers in the two clusters are further in line with the numbers reported for serial patient-derived O157:H7 isolates that underwent short-term microevolutionary changes (Rusconi et al., 2016). Taken together, the phylogenomics data identified the laboratory derived Nal^R^ mutant strains as the source of the researcher’s life-threatening disease rather than a conceivable ingestion of unrelated STEC O157:H7 isolates circulating at the same time. The results also clearly support a simultaneous co-infection with laboratory-housed strains, which were the causative agents of previous O157:H7 outbreaks. Co-infections have been only observed rarely in STEC (Rivas et al., 1993; Gilmour et al., 2007; Cheung et al., 2020), and the potential impact on disease manifestation by such mixed infections of strains featuring distinct genome and virulence traits has not been evaluated.

### Determination of Stx2a Production by ELISA and *Stx*2a Expression by qRT-PCR Following Exposure to the Antibiotics

**Figure 3:**
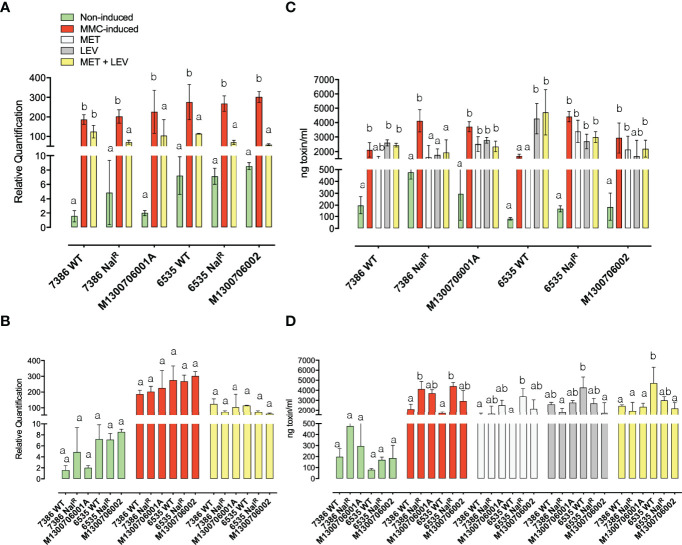
Stx-virulence phenotypes in response to hospital administered antibiotics Stx-production of the infection-associated laboratory and clinical isolates were recorded under non-induced, MMC-induced conditions and hospital administered antibiotics. Transcripts of s*tx_2a_* **(A, B)** were measured by RT-qPCR using two biological and technical replicates and normalized against the endogenous gene *tufA*. Values are shown in relative quantitation (RQ), after using the comparative C_T_ (ΔΔC_T_) method (Livak and Schmittgen, 2001). Stx_2_-production **(C, D)** was quantified by ELISA. Error bars depict standard deviation of two biological replicates. Statistical significance of the RT-qPCR and ELISA for each individual strain when comparing conditions **(A, C)**, and for each condition when comparing strains is shown **(B, D)**. Different letters among conditions in each strain **(A, C)** and among strains in each condition **(B, D)** indicate statistically significant differences.

We recorded production traits of Stx2a, the most potent allelic subtype associated with human disease (Tesh et al., 1993; Orth et al., 2007; Fuller et al., 2011), in response to MET and LEV in *wt* strains, laboratory-derived Nal^R^ mutants, and clinical isolates. For a comprehensive readout, we recorded both toxin transcript and protein levels under spontaneous (non-induced) and MMC-inducing conditions (**Figure 3**). Expression and production levels of the *stx*2a gene and Stx2 were measured by RT-qPCR (**Figures 3A, B**) and ELISA (**Figures 3C, D**), respectively. MMC is a potent Stx2 prophage-inducing agent that triggers toxin production *via* the SOS response mechanism (Raya and H’bert, 2009; Pacheco and Sperandio, 2012; Allué-Guardia et al., 2014). Bacterial counts (log CFU/ml) after 18 h of growth at 37°C in TSB medium alone were very similar among the six strains analyzed (**Table 2**). Statistically significant reductions in CFU/ml, compared to TSB, were observed in the presence of MET in 7386 *wt*, 7386 Nal^R^, 6535 *wt*, and patient isolates M1300706001A and M1300706002 (**Table 2**). The addition of both MET and LEV to the cultures resulted in statistically significant reductions in patient-derived strain M1300706001A and in 7386 Nal^R^ compared to counts observed in TSB alone (**Table 2**). LEV had no significant effect on the reduction of CFU/ml in any strain compared to TSB (**Table 2**). Therefore, the reduction in bacterial growth observed in cultures with the combination of MET and LEV were primarily due to MET.

**Table 2:** Colony counts of the different strains.

Strain	TSB	TSB+MMC	TSB+Metronidazole (MET)	TSB+Levofloxacin (LEV)	TSB+MET+LEV
7386 *wt*	8.9 ± 0.1^a^	8.2 ± 0.5^ab^	6.8 ± 0.3^b^	8.8 ± 0.5^a^	7.2 ± 0.5^ab^
7386 *nal* ^R^	8.7 ± 0.1^a^	8.1 ± 0.1^ab^	7.2 ± 0.4^b^	8.7 ± 0.1^a^	7.2 ± 0.4^b^
M1300706001A	8.9 ± 0.1^a^	8.5 ± 0.3^ab^	7.2 ± 0.2^b^	8.7 ± 0.2^a^	7.0 ± 0.4^b^
6535 *wt*	9.0 ± 0.1^a^	8.5 ± 0.1^ab^	7.1 ± 0.1^b^	9.0 ± 0.0^a^	8.3 ± 0.1^ab^
6535 *nal* ^R^	8.9 ± 0.1^ab^	8.4 ± 0.1^ab^	7.5 ± 0.5^b^	9.1 ± 0.1^a^	7.7 ± 05^ab^
M1300706002	9.0 ± 0.0^b^	8.6 ± 0.1^ab^	7.3 ± 0.3^a^	8.9 ± 0.1^b^	7.4 ± 0.2^ab^

Log [CFU/ml] of the different strains (average ± standard deviation) after 18 h growth in TSB, mitomycin, LEV, MET, and MET and LEV combined. Statistics are based on non-parametric Kruskal Wallis Anova test and Dunn’s multiple comparison test. Different letters in the same row among columns indicate statistically significant differences.

Several biotic and abiotic cues can induce the Stx converting prophage complement in STEC hosts, including antibiotics (Pacheco and Sperandio, 2012). Therapeutic use of antibiotics for STEC infections is thus controversial, as certain antibiotics are known to induce Stx2 phages and consequently toxin production through SOS-dependent activation of the phage lytic cycle (Kimmitt et al., 2000; Wong et al., 2000; Zhang et al., 2000; McGannon et al., 2010). Elevated toxin levels and *stx*2a transcripts were observed when compared to spontaneous conditions after exposure to MET and LEV (**Tables 3**, **4**). Significantly increased production of Stx2 was observed in each of the six strains analyzed with combined MET and LEV compared to TSB (p<0.05), except for 7386 Nal^R^, which showed high levels of toxin production in the presence of TSB alone. The highest Stx2 production with combined MET and LEV was observed in 6535 *wt* (average 4717 ng toxin/ml) with significantly higher Stx2 production compared to 7386 *wt*, 7386 Nal^R^, and both patient isolates (p<0.05) (**Figure 3D**). Significantly higher production of Stx2 was observed in 6535 *wt* compared to the 6335 Nal^R^ patient isolate M1300706002 (**Table 3**). The 6535 *wt* strain produced significantly less Stx2 under MET-induction when compared to the combination of the two antibiotics.

**Table 3:** Stx2a production of the different strains.

Strain	TSB	TSB+MMC	TSB+Metronidazole (MET)	TSB+Levofloxacin (LEV)	TSB+MET+LEV
7386 *wt*	197.9 ± 74.7^a^	2110.5 ± 495.6^b^	1186.0 ± 483.2^ab^	2594.0 ± 200.6^b^	2440.0 ± 117.9^b^
7386 *nal^R^*	477.0 ± 56.4^a^	4122.1 ± 777.8^b^	1606.8 ± 826.1^a^	1767.3 ± 423.5^a^	1934.8 ± 858.7^a^
M1300706001A	295.9 ± 226.8^a^	3709.5 ± 353.5^b^	2507.8 ± 506.1^b^	2773.1 ± 205.0^b^	2352.9 ± 358.3^b^
6535 *wt*	80.3 ± 10.7^a^	1686.6 ± 139.3^a^	1149.7 ± 258.6^a^	4272.7 ± 1056.8^b^	4717.0 ± 1579.7^b^
6535 *nal^R^*	169.8 ± 25.7^a^	4413.6 ± 363.2^b^	3386.8 ± 775.0^b^	2695.4 ± 496.8^b^	2988.4 ± 378.8^b^
M1300706002	185.7 ± 116.8^a^	2935.4 ± 1035.1^b^	2140.9 ± 909.7^b^	1683.4 ± 1087.5^ab^	2193.3 ± 579.1^b^

Stx2a production [ng/ml] in the different strains (average ± standard deviation) after 18 h growth in TSB, mitomycin, LEV, MET and MET+LEV combined. Statistics are based on two-way ANOVA and Tukey’s multiple comparison tests. Different letters in the same row among columns indicate statistically significant differences.

**Table 4:** *stx*2a gene expression of the different strains.

Strain	TSB	TSB+MMC	TSB+MET+LEV
7386 *wt*	1.6 ± 0.8^a^	186.9 ± 24.4^b^	124.6 ± 32.4^b^
7386 *nal^R^*	4.9 ± 4.5^a^	202.4 ± 34.3^b^	70.7 ± 10.0^a^
M1300706001A	2.0 ± 0.4^a^	226.0 ± 110.2^b^	104.4 ± 81.6^a^
6535 *wt*	7.2 ± 2.6^a^	275.9 ± 89.6^b^	114.1 ± 2.2^a^
6535 *nal^R^*	7.1 ± 1.1^a^	268.2 ± 39.5^b^	69.8 ± 10.8^a^
M1300706002	8.5 ± 0.5^a^	302.0 ± 27.6^b^	58.2 ± 4.9^a^

qPCR results for stx2a gene expression in the different strains (average ± standard deviation) after 18 h growth in TSB, MMC, and LEV+MET combined. Statistics are based two-way ANOVA and Tukey’s multiple comparison tests. Different letters in the same row among columns indicate statistically significant differences.

Stx2 production levels with exposure to MMC were higher than those induced by combined MET and LEV in the two Nal^R^ mutant strains and in the two patient isolates (**Table 3**), but statistically significantly increased Stx2 production was only observed in 7386 Nal^R^. Lower Stx2 production by MMC was observed in both *wt* strains, with significantly lower production in 6535 *wt* compared to exposure to LEV and the combination of MET and LEV.

Stx2 transcript levels were examined only in the presence of LEV and MET combined, and not with the antibiotics individually (**Figures 3A, B** and **Table 4**). Results indicated that the induction effect of these antibiotics was not as pronounced as in samples treated with MMC. Results with MET+LEV treatments were not significantly different than treatment with TSB alone with the exception of strain 7386 *wt*. Another notable exception was that results with strain 7386 *wt* did not show a statistical difference between MMC and MET+LEV treatments.

When comparing *stx*2 expression versus Stx2 production results, a similar trend was observed in most strains. 7386 *wt* showed no significant differences in both expression and production of toxins in MMC versus MET+LEV, while the other strains showed significant differences in both toxin expression and production in MMC versus MET+LEV (**Tables 3**, **4**). Host derived strains M1300706001A/7386-Nal^R^ and M1300706002/6535-Nal^R^ showed significant differences in *stx*2a gene expression under MMC versus MET+LEV conditions, but not in toxin production. This may indicate a delayed production of toxins in this strain; however, this would require further study. We note here a disconnection between transcript and Stx2 protein levels has been previously reported in O157:H7 strains, though the reason for this apparent lack of correlation remains to be elucidated (Leenanon et al., 2003; Neupane et al., 2011).

## Conclusions

High-resolution genomic epidemiology techniques have been extensively used in outbreak investigations to identify the contaminated source and emergence of hypervirulent STEC lineages (Underwood et al., 2013; Amigo et al., 2015; Dallman et al., 2015a; Dallman et al., 2015b; Eppinger and Cebula, 2015). Assuring a timely and informed response in the control of microbial outbreaks is challenging, and techniques with high discriminatory power become of particular importance to distinguish outbreak isolates that form tight clonal complexes with only few genetic polymorphisms. Particularly, *de novo* SNP typing complements often surpass other more labor-intensive molecular typing schemes that have been developed for STEC pathogen populations over the last decades (Sadiq et al., 2014). Our investigation of this clinical STEC case identified a laboratory contamination with an Stx_2a_-positive O157:H7 strain mixture as the source of a severe human infection. PFGE in combination with SNP profiling allowed us to establish a “close” clonal relationship of the compared laboratory *wt*/NalR-mutant and patient strains reflecting the short-term microevolutionary changes in their genomes. The detected SNP numbers are in line with reports by us and other groups for serial outbreak isolates of O157:H7 (Dallman et al., 2015b; Rusconi et al., 2016; Dallman et al., 2021). Unfortunately, to date no effective treatment or prophylaxis for HUS is known (Goldwater and Bettelheim, 2012). Certain antibiotics are known to mobilize Stx_2a_-phages and ultimately cause toxin production, thus the therapeutic use of antibiotics remains highly controversial (Mead and Griffin, 1998; Rahal et al., 2015; Freedman et al., 2016). The treatment regimen of the patient provided us with the unique opportunity to assess the impact of the individual administered antibiotics or cocktails on the strains’ Stx production capabilities. *In vitro* assays showed that exposure to MET and LEV likely increased the pathogenic potential of the infective strains. In sublethal doses, these antibiotics elevated both toxin-transcript and -production levels in this clinical case, and in consequence may have exacerbated the symptoms and the severity of the disease. As demonstrated in this study, the integration of genome and virulence information is critical for outbreak investigations and improved risk assessment of STEC (Sadiq et al., 2014; Eppinger and Cebula, 2015), and our findings call for awareness of increased Stx production capabilities following the therapeutic treatment with antibiotics.

## Accession Numbers

The sequencing datasets for all isolates analyzed in this study have been deposited in the Sequence Read Archive (SRA) and the Whole Genome Shotgun Repository at National Center for Biotechnology Information (NCBI) under BioProjects PRJNA407949 and PRJNA750123. Accessions for reads, assembled, and annotated draft genomes, along with strain-associated metadata are provided in **Supplementary Table 2**.

## Data Availability Statement

The datasets presented in this study can be found in online repositories. The names of the repository/repositories and accession number(s) can be found in the article/supplementary material.

## Author Contributions

Conceived and designed the experiments, ME and PF. Analyzed the data, ME, SA, AA-G, LB, JG, and PF. Sequence assemblies and submissions to the NCBI repositories, AK and ME. Wrote the paper, ME, SA, and PF. All authors contributed to the article and approved the submitted version.

## Funding

Research reported in this publication was supported by the National Institute of General Medical Sciences of the National Institutes of Health under Award Numbers [SC1GM135110] and the US Department of Homeland Security [2014-ST-062-000058] to ME. The content is solely the responsibility of the authors and does not necessarily represent the official views of the National Institutes of Health or the US Department of Homeland Security. The mention of a trade name, proprietary product, or specific equipment does not constitute a guarantee or warranty by the USDA and does not imply approval to the exclusion of other products that might be suitable. The USDA and FDA are an equal opportunity employer and provider. SA co-prepared this article in her personal capacity before joining FDA. The opinions expressed in this article are the author’s own and do not reflect the view of the FDA, the Department of Health and Human Services or the United States government.

## Author Disclaimer

The portion of this manuscript prepared by SA was done in her personal capacity before joining FDA. The opinions expressed in this article are the author’s own and do not reflect the view of the FDA, the Department of Health and Human Services or the United States government.

## Conflict of Interest

The authors declare that the research was conducted in the absence of any commercial or financial relationships that could be construed as a potential conflict of interest.

## Publisher’s Note

All claims expressed in this article are solely those of the authors and do not necessarily represent those of their affiliated organizations, or those of the publisher, the editors and the reviewers. Any product that may be evaluated in this article, or claim that may be made by its manufacturer, is not guaranteed or endorsed by the publisher.

## Supplementary Material

Supplementary Table 1 PCR-primers and -cycling conditions.

Supplementary Table 2 Genomes and associated metadata of strains used in this study.

Supplementary Table 3 Virulence profiles of strains 7386 and 6535.

Supplementary Table 4 Catalogued core genome SNPs.
