# Supplementary material for: Genome Sequence Analysis and Characterization of Shiga Toxin 2 Production by Escherichia coli O157:H7 Strains Associated With a Laboratory Infection
Source: Front Cell Infect Microbiol. 2022 Jun 13;12:888568. doi: 10.3389/fcimb.2022.888568 (PMC9234449; doi:10.3389/fcimb.2022.888568)
Supplement: Supplementary Table 1 — PCR-primers and -cycling conditions. [file Table_1.docx]

**Table S1.** PCR-primers and -cycling conditions

| Primer | Sequence 5’- 3’ | PCR program | Amplicon size | Reference |
| --- | --- | --- | --- | --- |
| GyrA-F | ACCGGTCAACATTGAGGAAG | Denaturation: 98°C 30s  Annealing: 60°C 30s  Elongation: 72°C 60s | 953 bp | This study |
| GyrA-R | GCAACTGGGTCTGGGAGTAG |  |  | This study |
| Top-F | CTGAACTGCTGGCGGAGAT | Denaturation: 98°C 30s  Annealing: 63°C 30s  Elongation: 72°C 60s | 784 bp | This study |
| Top-R | GCCATGTCGCCTTTCTCTT |  |  | This study |
| stx2-F | TCCCGTCAACCTTCACTGTA | Denaturation: 95°C 15s  Annealing/Extension: 60°C 60s  Melting curve: 60-95°C | 115 bp | (Wang et al., 2002) |
| stx2-R | GCGGTTTTATTTGCATTAGC |  |  | (Wang et al., 2002) |
| tufA-for | TGGTTGATGACGAAGAGCTG | Denaturation: 95°C 15s  Annealing/Extension: 60°C 60s  Melting curve: 60-95°C | NA | (Gobert et al., 2007) |
| tufA-rev | GCTCTGGTTCCGGAATGTAA |  |  | (Gobert et al., 2007) |
